# Patient Awareness of Soft-Tissue Irritants After Placement of Porcelain Laminate Veneers

**DOI:** 10.7759/cureus.30434

**Published:** 2022-10-18

**Authors:** Esam H Mohammed Dhaifullah, Maram S Zarnoog, Musab A Muqaybil, Abdulaziz F Alrogi, Shoug M Albugami

**Affiliations:** 1 Preventive Dental Science, Vision College of Dentistry and Nursing, Riyadh, SAU; 2 Dentistry, Basmatic Al Gmaila for Dentistry and Dermatology, Riyadh, SAU

**Keywords:** dentistry, soft-tissue irritation, porcelain, veneers, porcelain laminate veneers

## Abstract

Background

Porcelain is considered the most esthetic and biocompatible material in dentistry with the ability to imitate sound enamel. Research into porcelain laminate veneers has focused mainly on technical or aesthetic aspects, rather than the reaction of the surrounding soft tissues. This study aims to evaluate the knowledge, attitudes, and practice regarding patient maintenance of soft-tissue alternation occurring after the placement of veneers.

Methodology

A descriptive, cross-sectional survey was chosen for this study to identify patient gingival alternation that occurred after the placement of veneers. Statistical analysis was performed using R version 3.6.3. Counts and percentages were used to summarize the study variables. The chi-square test was used to assess the association between categorical variables. Adverse events included hyperplasia, gum recession, change in color, change in taste/smell, toothache, and redness. Spearman’s correlation was used to assess the association between hygiene, awareness, and the number of adverse events experienced after installing the veneers. Hypothesis testing was performed at a 5% level of significance.

Results

The study questionnaire was completed by 98 respondents. Males and females represented 34.7% and 65.3% of the study sample, respectively. One-half (53.1%) of the respondents were 30-40 years old, and one-quarter were 41-50 years old. Only one-half of the respondents were satisfied with the final result after installing the veneers (53.1%). Satisfaction was not significantly different between males and females (p = 0.818). Results showed better awareness and dental hygiene in females. Only 11.8% of the males reported visiting the dentist due to gingival problems before installing the veneers compared to 35.9% of the females (p = 0.021). The most common problems reported by the respondents were hyperplasia (62.2%) and a change in smell/taste (66.3%). Other common problems included color change (58.2%), toothache (59.2%), and redness (55.1%). Satisfaction was significantly higher in patients who did not experience gingival hyperplasia (70.3%) than in those who did (42.6%). Experiencing toothache and redness in the gums surrounding the lenses were associated with lower satisfaction (p < 0.001). The use of antibiotics was not associated with satisfaction (p = 0.495).

Conclusions

Our study indicated a low level of awareness and satisfaction with porcelain laminate veneer placement. There was a statistically significant association between dental hygiene and awareness scores. Dental hygiene was also positively associated with satisfaction with dental veneers. A lower awareness score was associated with lower satisfaction. Further, higher awareness was associated with higher satisfaction.

## Introduction

In dentistry, porcelain is regarded as the most aesthetically pleasing and biocompatible material because it can mimic healthy enamel [1]. Research into porcelain laminate veneers has focused mainly on technical or aesthetic aspects, rather than the reaction of the surrounding soft tissues. Previous research suggests that the marginal gingival tissues should not react at all, or even positively, to porcelain veneers [1]. Because gingival health affects clinical performance, studies have measured and investigated gingival recession, pocket depth, and gingival index (GI) in relation to porcelain laminate veneer treatment [2,3]. Regarding the margin placement, finish, and polish of the restorations, studies have investigated the impact of various restorations on gingival health [4-6].

Patients are always concerned about their smiles and oral health. Lately, with veneers, it is possible to create excellent esthetic results and yet retain considerable solid tooth structure while maintaining soft-tissue health. Successful results of porcelain laminate veneers depend not only on the clinical and laboratory technique used for veneer fabrication but also on patient awareness regarding maintaining excellent soft-tissue health [6].

In this study, we aimed to evaluate the knowledge, attitudes, and practice regarding patient maintenance of soft-tissue alternation occurring after the placement of veneers.

## Materials and methods

A descriptive, cross-sectional survey was chosen for this study to identify patient gingival alternation that occurred after veneer placement. The awareness of patients toward the maintenance phase and how they followed the dentist’s instructions regarding cleaning the veneers and the gingiva was also assessed.

All patients of both genders who had dental veneers during the study duration, were older than 20 years, and were willing to participate in the study were included. Patients with a systemic disease, pregnant or intending to become pregnant, heavy smokers, and patients with poor oral hygiene were excluded.

R version 3.6.3 was used to conduct the statistical analysis. We compiled the study’s variables using counts and percentages. The chi-square test was employed to evaluate the relationship between categorical variables. The correlation between the number of negative reactions to veneers and satisfaction was evaluated using the chi-square test for linear trend. Dental hygiene was assessed using one question. Awareness regarding dental veneers was assessed using the following four questions: (1) the dentist explained the pros and cons of the procedure; (2) the dentist explained the correct dental hygiene routine after installing the veneers; (3) followed the doctor’s instructions regarding dental hygiene; (4) attended dental and gum cleaning appointments after installing the veneers.

Adverse events included hyperplasia, gum recession, change in color, change in taste/smell, toothache, and redness. Spearman’s correlation was used to assess the association between hygiene, awareness, and the number of adverse events experienced after installing the veneers. Hypothesis testing was performed at a 5% level of significance.

## Results

The study questionnaire was completed by 98 respondents. The sociodemographic characteristics of the respondents are shown in Table 1.

**Table 1:** Descriptive statistics for the study sample.

	N = 98
Age (years)	
18–29	19 (19.4%)
30–40	52 (53.1%)
41–50	23 (23.5%)
50 or more	4 (4.08%)
Gender	
Female	64 (65.3%)
Male	34 (34.7%)
Education	
University or higher	73 (74.5%)
High school or less	25 (25.5%)
Marital status	
Married	72 (73.5%)
Single	26 (26.5%)
Health problems	
No	87 (88.8%)
Yes	11 (11.2%)
Daily frequency of teeth brushing	
One time	40 (40.8%)
Two times	40 (40.8%)
Three times	17 (17.3%)
Four or more times	1 (1.02%)
Smoker	
No	76 (77.6%)
Yes	22 (22.4%)
Smoking frequency	
1–10	7 (31.8%)
11–20	10 (45.5%)
More than 20	5 (22.7%)
Habits that can hurt teeth	
Breathing through the mouth	14 (14.3%)
Bruxism	15 (15.3%)
Nail biting	4 (4.08%)
None	55 (56.1%)
Occlusal problems	10 (10.2%)

Males and females represented 34.7% and 65.3% of the study sample, respectively. One-half (53.1%) of the respondents were 30-40 years old, and one-quarter were 41-50 years old. Three-quarters of the respondents completed a university education (74.5%), and three-quarters (73.5%) were married. Only 11.2% of the respondents had health problems. All respondents reported brushing their teeth at least once. Smokers represented 22.4% of the study sample. Less than one-half of the respondents smoked 11-20 cigarettes, and 31.8% smoked less than 10 cigarettes daily. Reported harmful habits to the teeth included bruxism (15.3%), nail biting (4.08%), and occlusal problems (10.2%). More than one-half of the respondents did not report any problems (56.1%).

As shown in Table 2, the time since installing veneers did not vary between males and females (no significant difference; p = 0.193). More than one-third of the respondents had the veneers installed for less than one year, and 33.7 had it for two to three years. The remaining 14.3% and 11.2% reported having the veneers for four to five years and more than five years, respectively. Only one-half of the respondents were satisfied with the final result after installing the veneers (53.1%). Satisfaction was not significantly different between males and females (p = 0.818). Results showed better awareness and dental hygiene in females. Only 11.8% of the males reported visiting the dentist due to gingival problems before installing the veneers compared to 35.9% of the females (p = 0.021). A higher proportion of females (53.1%) reported cleaning the gums and between veneers than males (29.4%), and the difference was statistically significant (p = 0.042). More than one-third of the females reported having dental sensitivity (37.5%) compared to only 5.88% of the males (p = 0.002). Only one male (2.94%) knew the pros and cons of the procedure compared to 43.8% of the females (p < 0.001). Similarly, 8.82% of the males had the correct dental hygiene routine explained to them by the dentist compared to 51.6% of the females (p < 0.001). Compliance with the dentists’ instructions was higher in females (53.1%) than in males (20.6%). Only one-third of the males reported attending dental and gum cleaning appointments compared to 53.1% of the females, although the difference was not statistically significant (p = 0.233).

**Table 2:** Dental hygiene, awareness, and time since veneer installation. Data were summarized using counts and percentages.

	All	Female	Male	P-value
	N = 98	N = 64	N = 34	
Time since installing dental veneers				
1–12 months	40 (40.8%)	26 (40.6%)	14 (41.2%)	0.193
2–3 years	33 (33.7%)	21 (32.8%)	12 (35.3%)	
4–5 years	14 (14.3%)	7 (10.9%)	7 (20.6%)	
>5 years	11 (11.2%)	10 (15.6%)	1 (2.94%)	
Satisfied with the final result after installing veneers				
No	46 (46.9%)	29 (45.3%)	17 (50.0%)	0.818
Yes	52 (53.1%)	35 (54.7%)	17 (50.0%)	
Ever visited the dentist before installing the veneers due to gingival problems				
No	71 (72.4%)	41 (64.1%)	30 (88.2%)	0.021
Yes	27 (27.6%)	23 (35.9%)	4 (11.8%)	
Clean the gums and between veneers using dental floss or a water pump				
No	54 (55.1%)	30 (46.9%)	24 (70.6%)	0.042
Yes	44 (44.9%)	34 (53.1%)	10 (29.4%)	
Dental sensitivity before installing the lenses				
No	72 (73.5%)	40 (62.5%)	32 (94.1%)	0.002
Yes	26 (26.5%)	24 (37.5%)	2 (5.88%)	
The dentist explained the pros and cons of the procedure				
No	69 (70.4%)	36 (56.2%)	33 (97.1%)	<0.001
Yes	29 (29.6%)	28 (43.8%)	1 (2.94%)	
The dentist explained the correct dental hygiene routine after installing the lenses				
No	62 (63.3%)	31 (48.4%)	31 (91.2%)	<0.001
Yes	36 (36.7%)	33 (51.6%)	3 (8.82%)	
Followed the doctor’s instructions regarding dental hygiene				
No	57 (58.2%)	30 (46.9%)	27 (79.4%)	0.004
Yes	41 (41.8%)	34 (53.1%)	7 (20.6%)	
Attended dental and gum cleaning appointments after installing the lenses				
No	51 (52.0%)	30 (46.9%)	21 (61.8%)	0.233
Yes	47 (48.0%)	34 (53.1%)	13 (38.2%)	

**Figure 1:**
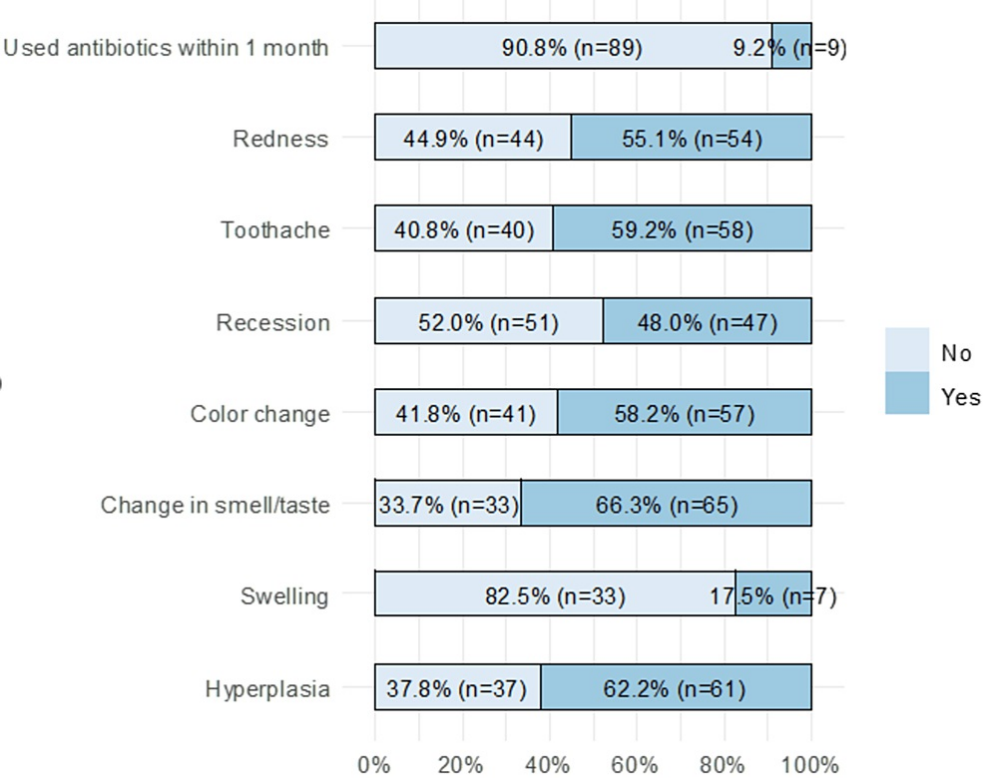
Problems related to dental veneers.

The most common problems reported by the respondents were hyperplasia (62.2%) and change in smell/taste (66.3%). Other common problems included color change (58.2%), toothache (59.2%), and redness (55.1%) (Figure 1).

Results showed that veneer-related problems were associated with satisfaction (Table 3). Satisfaction was significantly higher in patients who did not experience gingival hyperplasia (70.3%) than in those who did (42.6%). After installing the veneers, change in color, gum recession around the lenses, and bad taste or smell were associated with lower satisfaction. Similarly, experiencing toothache and redness in the gums surrounding the lenses was associated with lower satisfaction (p < 0.001). The use of antibiotics was not associated with satisfaction (p = 0.495).

**Table 3:** Association between satisfaction and veneer-related problems. Data were summarized using counts and percentages. Statistical analysis was performed using the chi-square test of independence.

	Satisfaction		P-value
	No	Yes	
	N = 46	N = 52	
Gingival hyperplasia around the veneer			
No	11 (29.7%)	26 (70.3%)	0.014
Yes	35 (57.4%)	26 (42.6%)	
Swelling in the gums (pregnancy)			
No	13 (39.4%)	20 (60.6%)	0.691
Yes	2 (28.6%)	5 (71.4%)	
Bad smell or taste after installing the veneer			
No	7 (21.2%)	26 (78.8%)	0.001
Yes	39 (60.0%)	26 (40.0%)	
Change in the color veneer			
No	13 (31.7%)	28 (68.3%)	0.018
Yes	33 (57.9%)	24 (42.1%)	
Gum recession around the veneer			
No	11 (21.6%)	40 (78.4%)	<0.001
Yes	35 (74.5%)	12 (25.5%)	
Toothache			
No	8 (20.0%)	32 (80.0%)	<0.001
Yes	38 (65.5%)	20 (34.5%)	
Redness in the gums surrounding the veneer			
No	9 (20.5%)	35 (79.5%)	<0.001
Yes	37 (68.5%)	17 (31.5%)	
Used antibiotics during the past month			
No	43 (48.3%)	46 (51.7%)	0.495
Yes	3 (33.3%)	6 (66.7%)	

**Figure 2:**
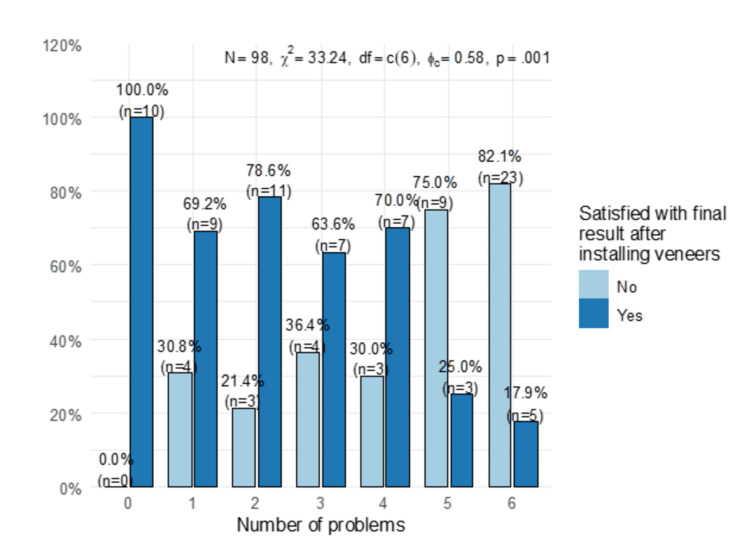
Association between veneer-related adverse events and satisfaction.

A statistically significant association was observed between veneer-related problems and satisfaction with the results (p = 0.001). The highest satisfaction was observed when no adverse events were experienced, and the lowest was observed when five or six adverse events were experienced (Figure 2).

A statistically significant association was noted between dental hygiene and awareness score (r = 0.416, p < 0.001), indicating that better dental hygiene is associated with a better awareness score (Table 4). Dental hygiene was also positively associated with satisfaction with dental veneers (r = 0.315, p < 0.01) and negatively associated with the number of dental-related problems experienced (r = -0.354, p < 0.001). Lower awareness score was associated with higher number of adverse events experienced (r = -0.513, p < 0.001) and lower satisfaction (r = -0.543, p < 0.001). Higher awareness was associated with higher satisfaction (r = 0.305, p < 0.01).

**Table 4:** Association between awareness, dental hygiene, and veneer-related problems. Correlation computed using Spearman’s correlation with listwise deletion. * p < 0.05, ** p < 0.01, *** p < 0.001.

	Dental hygiene	Number of adverse events	Awareness score
Clean gums			
Adverse events	-0.354^***^		
Awareness score	0.416^***^	-0.513^***^	
Satisfaction	0.315^**^	-0.543^***^	0.305^**^

## Discussion

Nowadays, the majority of people, particularly women, are concerned with their physical appearance. Therefore, determining the level of knowledge about laminate veneers is considered important in dental practice. The results of assessing the level of knowledge aid in determining the need for increased awareness [7].

Veneers are suggested for several dental conditions, such as tooth discoloration, diastema closure, cracked or chipped teeth, minor anterior tooth misalignments and rotations, teeth reshaping, large cervical lesions, labial surface caries, and amelogenesis imperfecta disorder. Regarding longevity, plaque buildup, and aesthetics, veneers perform better than resin composites. They are also widely accepted by patients and regarded as a secure option [8].

The development of the etched porcelain laminate veneer is the result of decades of research that culminated in the fusion of the acid-etch method and porcelain, the most traditional aesthetic material in dentistry. Glazed porcelain has several benefits, including abrasion resistance, biocompatibility with gingival tissues, long-term color stability, and aesthetic appeal [9]. The dentist’s innovative spirit is combined with the laboratory technician’s expertise in ceramics to produce aesthetically pleasing and predictable results. Veneers made in laboratories have numerous inherent benefits, such as their relative thinness (0.4-0.6 mm), exact fit, anatomical accuracy, gradient shading, and suitable surface texture. Overall, porcelain veneers are currently more attractive and durable than any direct or indirect resin substitute [9]. Dentinal sensitivity and the difficulty of repairing fractured veneers may be disadvantages, and periodontal problems may occur as a result of overcontouring of the veneers [7].

Many studies have investigated the longevity of porcelain veneers. In our study, more than one-third of the respondents had veneers installed for less than one year, and 33.7 had it for two to three years. The remaining 14.3% and 11.2% reported had veneers for four to five years and more than years, respectively. In a retrospective clinical study, Beier et al. [10] reported a five-year survival rate of 94.4% and a 10-year survival rate of 93.5%. Similar findings were reported in a randomized clinical trial by Layton and Walton [11]. The study reported a 10-year survival rate of 96% and a 20-year survival rate of 91%. Additionally, over a seven-year period, Smales and Etemadi [12] reported a 95% survival rate for porcelain veneers. It is important to emphasize that these studies used a strict assessment of remaining enamel and bonding systems, just like other studies that reported a high survival rate of porcelain veneers. To ensure predictable outcomes, careful, conservative preparation and optimal isolation during cementation are needed.

Because aesthetic satisfaction is a personal experience, it is a complicated process. Patient satisfaction, however, may be significantly influenced by several variables, including the longevity of the final aesthetic result, the amount of tooth preparation necessary for the chosen material type, and the cost of the procedure [13].

Many clinical studies that evaluated the longevity of porcelain veneers considered patients’ satisfaction with the treatment, and the range of satisfaction in these studies was 80-100% [14-16]. Our study reported fewer figures as only one-half of the respondents were satisfied with the final result after installing the veneers (53.1%). Satisfaction was not significantly different between males and females (p = 0.818). After two years, Meijering et al. [17] compared how patients responded to feldspathic porcelain, direct composite, and indirect composite veneer restorations. Patients responded most positively to porcelain veneers (93%), followed by indirect composite veneers (82%), and direct composite veneers (67%). The response of patients to composite veneers and porcelain veneers was not statistically different, according to Nalbandian and Millar (2009) [13]. These two studies could be biased because the level of preoperative discoloration or malposition can influence the postoperative degree of transformation and, in turn, the patient’s reaction.

In our study, the most common problems reported by the respondents were hyperplasia (62.2%) and change in smell/taste (66.3%). Other common problems included color change (58.2%), toothache (59.2%), and redness (55.1%). Granell-Ruiz et al. [18] reported that recessions were observed in 7.7% of the teeth treated. Further, several patients complained of sensitivity in the teeth that were treated with this type of restoration during the first few days after it was placed, but such sensitivity seemed to gradually disappear over time. Recessions were noted in 30% of cases in the study by Dumfahrt et al. [19], which was justified by stating that recessions are common in patients with good oral hygiene and that the proportion of people with recessions rises with age. Some patients continued to express sensitivity, which can be explained by the fact that the cutting was more aggressive in these patients because the dental malposition was the reason they had visited the clinic in the first place. Given that none of these patients had any alterations or filtrations, this could possibly be the cause. None of the patients expressed concerns about sensitivity at the time of the revision, according to other clinical studies carried out by other authors [20,21]. In other studies [14,22], pigmentation was produced in 22% and 25% of the restorations, and even much higher in other studies [20], where the pigmentation only appeared in 7% of the restorations. Granell Ruiz et al. [18] found that 39.3% of the marginal pigmentation observed was slightly higher than the results of those studies.

Our study showed a statistically significant association between dental hygiene and awareness score, indicating that better dental hygiene is associated with a better awareness score. Dental hygiene was also positively associated with satisfaction with dental veneers. A lower awareness score was associated with lower satisfaction. In addition, higher awareness was associated with higher satisfaction. In Saudi Arabia, Alharbi et al. [23] reported that the population’s knowledge of laminate veneers, their uses, care, side effects, and real indications appeared to be insufficient. Individuals who were current users of laminate veneers did not significantly differ in their knowledge of veneers regarding side effects, lifespan, and proper care and cleaning of veneers. According to Nalbandian and Millar [24], full knowledge of side effects and care for laminate veneers was associated with significantly higher rates of satisfaction post-treatment, emphasizing the importance of patient education prior to the insertion of dental veneers.

## Conclusions

Our study indicated a low level of awareness and satisfaction with porcelain laminate veneer placement. There was a statistically significant association between dental hygiene and awareness score. Dental hygiene was also positively associated with satisfaction with dental veneers. A lower awareness score was associated with lower satisfaction. In addition, higher awareness was associated with higher satisfaction.
